# Chronic General Peritonitis

**Published:** 1882-03

**Authors:** 


### CHRONIC GENERAL PERITONITIS.

In the December number of the New York *Medical Journal and Obstetrical Review*, Dr. Alfred L. Carroll, of New Brighton, New York, records an interesting case of chronic general peritonitis, which seemed to have taken its origin in an old pleurisy on the left side, the inflammation passing through the diaphragm and causing at first a perihepatitis, or perhaps an intermediate perisplenitis. The case exemplifies a condition that has been classed among the curiosities of medical experience, for chronic general peritonitis, independent of tuberculosis or carcinosis, is either ignored or its existence denied by most writers, save as a protraction of an acute purulent attack, and the few who recognize its existence differ as to its pathology and clinical history.

